# *sic-4* Reports in sick! Loss of SICKLE induces salicylic acid-dependent cell death in *Arabidopsis*

**DOI:** 10.1093/plphys/kiad237

**Published:** 2023-04-18

**Authors:** Moona Rahikainen

**Affiliations:** Plant Physiology, American Society of Plant Biologists, USA; Organismal and Evolutionary Biology Research Programme, Faculty of Biological and Environmental Sciences, University of Helsinki, FI-00014 Helsinki, Finland

Programmed cell death (PCD) is a central defense strategy in plants that allows the plant to eliminate stress-damaged tissues and limit the systemic spread of pathogens. PCD is regulated by a complex signaling network of cell death–promoting signals and counteracting negative regulators. Salicylic acid (SA) is a central hormonal regulator that together with reactive oxygen species (ROS) and calcium signaling drives defense-associated PCD (Van Aken and Van Breusegem 2015; Koster et al. 2022). A variety of gene regulation mechanisms, ranging from transcription to the post-translational modification of proteins, control the process of PCD (Van Aken and Van Breusegem 2015; Huysmans et al. 2017).

*SICKLE* is a plant-specific single-copy gene that encodes a proline-rich protein (Zhan et al. 2012). Arabidopsis mutants deficient in SICKLE, namely *sic* mutants, show pleiotropic abnormalities involving alterations in plant development and hypersensitivity to abiotic stresses (Zhan et al. 2012; Marshall et al. 2016; Wu et al. 2022). Previous studies have shown that SICKLE contributes to RNA metabolism by affecting microRNA biogenesis and alternative splicing (Zhan et al. 2012; Marshall et al. 2016). Recently, Wu et al. (2022) proposed a mechanism whereby SICKLE interacts with RNA debranching enzyme 1 (DBR1) to promote the degradation of lariat intronic RNAs (lariRNAs). LariRNAs are byproducts of mRNA splicing that are either degraded or processed to form small regulatory RNAs. Thus, lariRNAs play important regulatory roles in eukaryotic cells as precursors for microRNAs, small interfering RNAs, and small nucleolar RNAs (Neil and Fairbrother 2019). Moreover, lariRNAs function as decoys for RNA-binding proteins and thus modulate mRNA stability (Li et al. 2016). Taken together, lariRNAs are emerging as important post-transcriptional regulators of gene expression (Li et al. 2016; Neil and Fairbrother 2019; Wu et al. 2022).

In this issue of *Plant Physiology*, Wu et al. (2023) demonstrate that loss of SICKLE affects mRNA metabolism and induces SA-dependent PCD in *Arabidopsis* leaves. Using histochemical staining by 3,3′-diaminobenzidine, an indicator of ROS levels, and trypan blue, an indicator of cell death, Wu et al. (2023) show that the Arabidopsis *sic-4* mutant exhibits accumulation of ROS and local cell death in leaves. Moreover, the observed cell death is accompanied by changes in the expression of SA-associated genes (Wu et al. 2023). Interestingly, these hallmarks of PCD are abolished in the *DBR1-OE/sic-*4 mutant that overexpresses the DBR1 enzyme that catalyzes lariRNA degradation. Thus, the authors conclude that the PCD in *sic-4* is caused by accumulation of lariRNAs (Wu et al. 2022, 2023).

By analyzing differentially expressed genes, Wu et al (2023) discovered that transcripts associated with defense and SA homeostasis and signaling are upregulated in *sic-4* mutants. Interestingly, further analysis revealed that the alternatively spliced genes in *sic-4* were largely different from the differentially expressed genes (Wu et al. 2023). The observed differences in the splicing of transcripts associated with PCD suggest that alternative splicing may be involved in the activation of PCD in the *sic-4* mutant (Wu et al. 2023). Based on these results, Wu et al. (2023) suspected that the accumulation of lariRNAs might regulate mRNA stability by disturbing their interactions with RNA-binding proteins (Li et al. 2016). Taking advantage of the transcriptional inhibitor cordycepin and RNA sequencing, the authors analyzed the decay of mRNAs in wild-type and *sic-4* mutant plants. Faster decay of transcripts characterized as negative regulators of cell death and SA levels was observed in the *sic-4* mutant (Wu et al. 2023). Therefore, Wu et al. (2023) suggest that the cell death in *sic-4* results from alternative splicing and changes in the mRNA decay.

**Figure 1:**
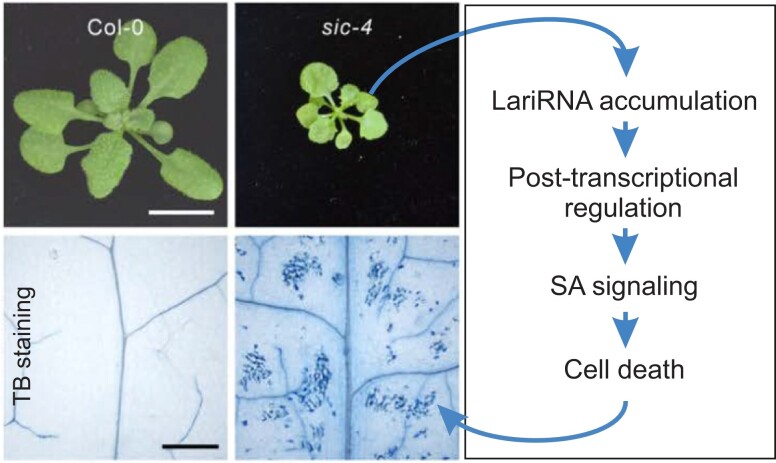
Diagram depicting the mechanism triggering cell death in Arabidopsis *sic-4* mutant. Lack of functional *SICKLE* induces hyperaccumulation of lariat RNAs (lariRNA). LariRNAs regulate gene expression at the post-transcriptional level activating SA signaling and programmed cell death (PCD) in *sic-4* leaves as visualized by trypan blue (TB), a cell impermeant stain used to estimate the number of dead cells in a population. Photographs of the wild-type and *sic-4* mutant plants and their leaves are adapted from Wu et al. (2023).

Finally, Wu et al. (2023) show that SA signaling is involved in the PCD observed in the *sic-4* mutant. The authors measured higher SA content and transcriptional activation of SA-responsive genes in the *sic-4* mutant compared with wild-type plants (Wu et al. 2023). In line with this, the cell death phenotype of *sic-4* was suppressed in *sic-4/sid2-1*, *sic-4/npr1-1*, and *sic-4/pad4*-1 double mutants that lack the SA biosynthesis enzyme SALICYLIC ACID INDUCTION DEFICIENT 2 (SID2) and SA signaling activators ENHANCED DISEASE SUSCEPTIBILITY 1 (EDS1) and PHYTOALEXIN DEFICIENT 4 (PAD4), respectively (Wu et al. 2023). The authors conclude that SA signaling functions downstream of changes in alternative splicing and mRNA decay in the *sic-4* mutant (Wu et al. 2023).

Plant stress responses and developmental processes have been extensively studied at the transcriptional level. However, our knowledge of the post-transcriptional regulation that shapes the transcriptome is deficient. Wu et al. (2022, 2023) have identified SICKLE as a post-transcriptional regulator that controls alternative splicing and mRNA stability via its interaction with DBR1. This research adds to our understanding of the factors regulating RNA metabolism in plants. In addition, the research by Wu et al. (2023) highlights the complexity of the regulatory network governing PCD in plants and introduces a novel mechanism, lariRNA hyperaccumulation, that triggers PCD (Fig. 1). Intriguingly, the authors hypothesize that PCD could be activated by a thus-far-unidentified nucleotide-binding site leucine-rich repeat protein that functions upstream of the EDS1-PAD4 signaling module and monitors the lariRNAs in plants (Wu et al. 2023). In the future, it will be interesting to study how SICKLE contributes to plant resistance to pathogen infection and if lariRNA-triggered PCD is a widespread defense mechanism in plants.

## Data Availability

No new data were generated or analyzed in this article.

## References

[kiad237-B1] Huysmans M , Lema S, Coll NS, Nowack MK. Dying two deaths—programmed cell death regulation in development and disease. Curr Opin Plant Biol. 2017:35:37–44. 10.1016/j.pbi.2016.11.005 27865098

[kiad237-B2] Koster P , DeFalco TA, Zipfel C. Ca^2+^ signals in plant immunity. EMBO J. 2022:41(12):e110741. 10.15252/embj.2022110741 35560235 PMC9194748

[kiad237-B3] Li Z , Wang S, Cheng J, Su C, Zhong S, Liu Q, Fang Y, Yu Y, Lv H, Zheng Y, et al Intron lariat RNA inhibits microRNA biogenesis by sequestering the dicing complex in *Arabidopsis*. PLoS Genet. 2016:12(11):e1006422. 10.1371/journal.pgen.1006422 27870853 PMC5147768

[kiad237-B4] Marshall CM , Tartaglio V, Duarte M, Harmon FG. The *Arabidopsis* sickle mutant exhibits altered circadian clock responses to cool temperatures and temperature-dependent alternative splicing. Plant Cell. 2016:28(10):2560–2575. 10.1105/tpc.16.00223 27624757 PMC5134976

[kiad237-B5] Neil C , Fairbrother WG. Intronic RNA: ad‘junk’ mediator of post-transcriptional gene regulation. Biochim Biophys Acta Gene Regul Mech. 2019:1862(11–12):194439. 10.1016/j.bbagrm.2019.194439 31682938 PMC7171924

[kiad237-B6] Van Aken O , Van Breusegem F. Licensed to kill: mitochondria, chloroplasts, and cell death. Trends Plant Sci. 2015:20(11):754–766. 10.1016/j.tplants.2015.08.002 26442680

[kiad237-B7] Wu C , Wang X, Zhen W, Nie Y, Li Y, Yuan P, Liu Q, Guo S, Shen Z, Zheng B, et al SICKLE Modulates lateral root development by promoting degradation of lariat intronic RNA. Plant Physiol. 2022:190(1):548–561. 10.1093/plphys/kiac301 35788403 PMC9434198

[kiad237-B8] Wu C , Zhen W, Wang X, Li Y, Wang W, Hu Z Deficiency of Arabidopsis SICKLE 1 triggers programmed cell death by disturbing alternative splicing and decay of mRNAs. Plant Physiol. 2023:192(3):2523–2536. doi: 10.1093/plphys/kiad192 PMC10315277 36974901

[kiad237-B9] Zhan X , Wang B, Li H, Liu R, Kalia RK, Zhu JK, Chinnusamy V. *Arabidopsis* proline-rich protein important for development and abiotic stress tolerance is involved in microRNA biogenesis. Proc Natl Acad Sci USA. 2012:109(44):18198–18203. 10.1073/pnas.1216199109 23071326 PMC3497810

